# Classification of Alzheimer's disease based on hippocampal multivariate morphometry statistics

**DOI:** 10.1111/cns.14189

**Published:** 2023-04-01

**Authors:** Weimin Zheng, Honghong Liu, Zhigang Li, Kuncheng Li, Yalin Wang, Bin Hu, Qunxi Dong, Zhiqun Wang

**Affiliations:** ^1^ Department of Radiology Aerospace Center Hospital Beijing China; ^2^ School of Medical Technology Beijing Institute of Technology Beijing China; ^3^ Department of Radiology Xuanwu Hospital of Capital Medical University Beijing China; ^4^ School of Computing, Informatics, and Decision Systems Engineering Arizona State University Tempe Arizona USA

**Keywords:** AD patient stratification, computer‐aided diagnosis, hippocampal morphometry, patch‐based feature selection, SVM classification

## Abstract

**Background:**

Alzheimer's disease (AD) is a neurodegenerative disease characterized by progressive cognitive decline, and mild cognitive impairment (MCI) is associated with a high risk of developing AD. Hippocampal morphometry analysis is believed to be the most robust magnetic resonance imaging (MRI) markers for AD and MCI. Multivariate morphometry statistics (MMS), a quantitative method of surface deformations analysis, is confirmed to have strong statistical power for evaluating hippocampus.

**Aims:**

We aimed to test whether surface deformation features in hippocampus can be employed for early classification of AD, MCI, and healthy controls (HC).

**Methods:**

We first explored the differences in hippocampus surface deformation among these three groups by using MMS analysis. Additionally, the hippocampal MMS features of selective patches and support vector machine (SVM) were used for the binary classification and triple classification.

**Results:**

By the results, we identified significant hippocampal deformation among the three groups, especially in hippocampal CA1. In addition, the binary classification of AD/HC, MCI/HC, AD/MCI showed good performances, and area under curve (AUC) of triple‐classification model achieved 0.85. Finally, positive correlations were found between the hippocampus MMS features and cognitive performances.

**Conclusions:**

The study revealed significant hippocampal deformation among AD, MCI, and HC. Additionally, we confirmed that hippocampal MMS can be used as a sensitive imaging biomarker for the early diagnosis of AD at the individual level.

## Graphical abstract

Hippocampal multivariate morphometry statistics can be used as a sensitive imaging biomarker to predict the development of Alzheimer's disease.

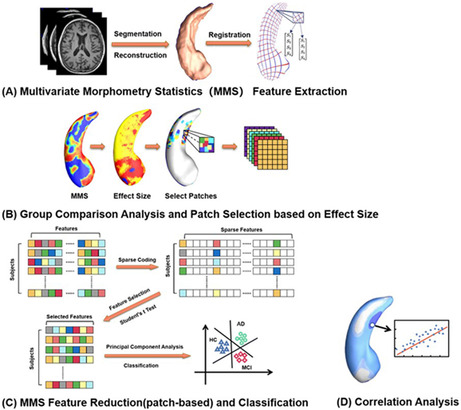

## 1 INTRODUCTION

Alzheimer's disease (AD) is the most prevalent cause of dementia, posing a significant threat to human health. Mild cognitive impairment (MCI) is considered as an intermediary stage between AD and normal cognitive function. Studies suggest that clinical intervention at this stage can be beneficial in delaying the onset of AD.^1^ Thus, it is of great theoretical significance and potential clinical application value to explore the pathogenesis of AD using new technologies and establish reliable imaging markers for early individual diagnosis of AD.^2^

Previous neuroimaging studies have highlighted the crucial role of the hippocampus in memory processes.^3^, ^4^, ^5^ Using various magnetic resonance imaging (MRI) methods, many studies have revealed AD‐associated hippocampal abnormalities, including volume atrophy,^6^, ^7^ hypometabolism,^8^ decreased spontaneous activation,^9^ and decreased functional connectivity.^10^, ^11^, ^12^, ^13^, ^14^, ^15^ Structural imaging can provide accurate anatomic structures up to 1 mm with relatively stable and repeatable results. In this study, we focused on structural analysis and attempted to capture subtle structural changes in the hippocampus during the early stages of AD.

Although numerous studies have focused on analyzing hippocampal volumes,^16^, ^17^, ^18^, ^19^ recent researches have suggested that surface‐based subregional structure analysis could offer advantages over volume measures.^20^, ^21^, ^22^, ^23^, ^24^, ^25^, ^26^ In the previous studies from our team,^11^, ^27^ we proposed a novel method to analyze the hippocampal surface deformations, which was multivariate morphometry statistics (MMS), including multivariate tensor‐based morphometry (mTBM) representing morphometry within surfaces and radial distance (RD) representing distances from the medial core to each surface point. By employing a group MMS analysis strategy, the differences in hippocampal morphometry deformations demonstrated stronger statistical power than volume measures.^11^, ^27^, ^28^, ^29^, ^30^ However, it has become increasingly important to derive a personalized accurate classification model for AD based on sensitive image features and advanced algorithms, rather than group analysis.^31^ In this study, we sought to search for a new biomarker to differentiate AD, MCI, and healthy controls using the hippocampal MMS analysis and support vector machine (SVM) method on individual levels.

In this work, based on MMS, we first studied hippocampal surface deformation in the AD, MCI, and HC groups, and identified the key subregions that showed significant differences among the three groups. Second, the SVM algorithm was used to construct a binary and triple‐classification model based on the selected MMS features. Finally, we examined correlations between regional hippocampal deformation and neuropsychological test scores to validate the clinical significance of the biomarker.

## 2 MATERIALS AND METHODS

### 2.1 Participants

Two hundred and forty right‐handed subjects participated in the study, including 75 AD patients, 63 MCI patients, and 102 HCs. The protocol was approved by the Medical Research Ethics Committee of Xuanwu Hospital and all subjects gave written informed consent in accordance with the Declaration of Helsinki.

All participants underwent a complete physical and neuropsychological assessment, including the mini‐mental state examination (MMSE), clinical dementia rating (CDR), and so on. The AD and MCI patients fulfilled the new research criteria for possible or probable AD and MCI.^32^

The controls fulfilled the following criteria: (a) no visual loss or hearing loss, as well as other neurological deficiencies; (b) no stroke, depression, or epilepsy, as well as other neurological or psychiatric disorders; (c) no abnormal findings in routine brain MRI; (d) no complaints about cognitive and memory; (e) CDR score of 0.

### 2.2 MRI acquisition

Magnetic resonance imaging examination was performed on a SIEMENS verio 3‐Tesla scanner (Siemens, Erlangen, Germany). The 3D T1‐weighted magnetization prepared rapid gradient echo (MPRAGE) sagittal images were performed with the following parameters: repetition time (TR)/echo time (TE)/inversion time (TI)/flip angle (FA) = 1900 ms/2.2 ms/900 ms/9°, image matrix = 256 × 256, slice number = 176, thickness = 1 mm.

### 2.3 Processing pipeline

**FIGURE 1:**
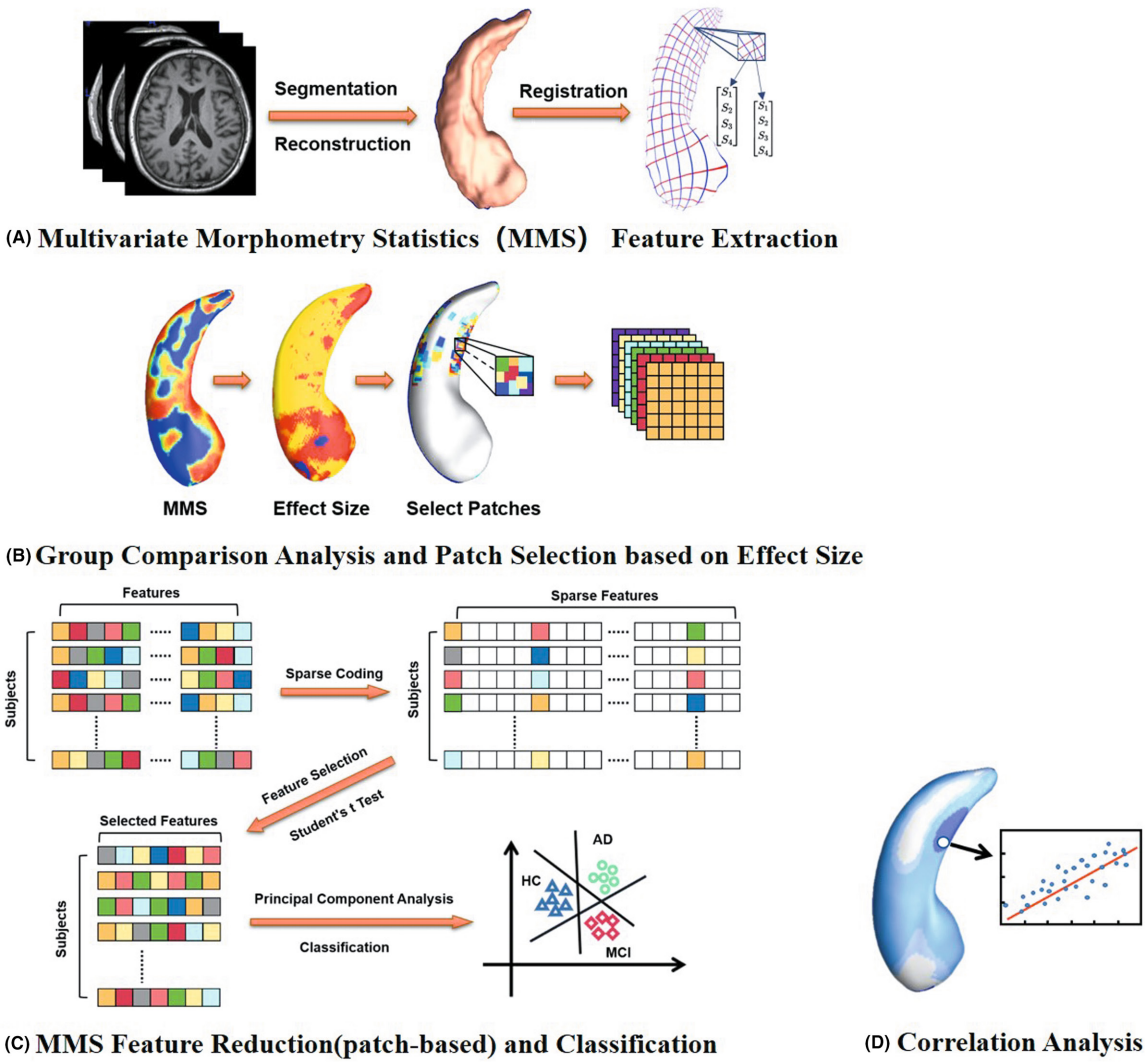
Whole processing pipeline is applied in this paper. First, we performed hippocampal multivariate morphometry statistics (MMS) feature extraction (A). Second, we made group comparison analysis of hippocampal MMS features and select patches based on effect Size (B). Third, based on the selected patches, we downscaled the MMS features and used it for subsequent classification (C). Finally, we made the Pearson correlation analysis between the MMSE scores and the MMS (RD and mTBM) features of hippocampus (D).

Figure 1 illustrates the whole processing pipeline applied in this paper, which includes the segmentation of hippocampus structures, surface reconstruction, surface registration, surface multivariate morphometry statistics (MMS) feature computation, group difference analysis, MMS feature dimension reduction, building SVM‐based binary and triple classifiers and the correlation analysis between MMS (RD and mTBM) features of subjects and their MMSE scores.

### 2.4 Hippocampus surface multivariate morphometry feature extraction and analysis

#### 2.4.1 Segmentation, reconstruction, and registration

We used FIRST, an integrated tool developed as a part of the FSL library, to automatically segment all the T1‐weighted brain volume MRI scans.^33^ These segmented images were spatially normalized into the MNI template space with a 9‐parameter (3 translations, 3 rotations, and 3 scales) linear transformation by using Minctracc algorithm^34^ for the correction of head tilt and alignment. To equalize image intensities across subjects, registered scans were also histogram‐matched. After segmentation for each individual hippocampus, the results were critically examined by two independent anatomists to verify their quality. The segmentation results of all hippocampi were then processed by surface conformal slit mapping.^35^ This approach allows us to efficiently analyze surface data over simpler parameter domains, which avoids the consideration of complex brain surfaces.^36^ Finally, we aligned surfaces in the parameter domain with a fluid registration technique to maintain a smooth, one‐to‐one topology.^37^, ^38^ The one‐to‐one correspondence achieved between vertices allows us to accurately analyze the local information on the hippocampal surface.

#### 2.4.2 Hippocampus surface multivariate morphometry statistics

To detect group differences in the subdivisions of hippocampus, some of the previously proposed features were used. The radial distance (RD) describes the morphological changes along the surface normal direction.^39^ Surface tensor‐based morphometry (TBM)^40^, ^41^, ^42^ examines the spatial derivatives (detJ, where J is the Jacobian matrix of the deformation from the registration) of the deformation maps that register each hippocampal surface to the common template. Suppose that $\varphi :S_{1}\to S_{2}$ is a map from surface *S*_1_ to surface *S*_2_. The derivative map of φ is the linear map between the tangent spaces $\mathrm{d\varphi}:\mathrm{TM}\left(p\right)\to \mathrm{TM}\left(\varphi \left(p\right)\right)$, induced by the map φ, which also defines the Jacobian matrix of φ. In the grid surface, the derivative map dφ is approximated by the linear map from one face $\left[v_{1},v_{2},v_{3}\right]$ to another $\left[w_{1},w_{2},w_{3}\right]$. First, the surfaces $\left[v_{1},v_{2},v_{3}\right]$ and $\left[w_{1},w_{2},w_{3}\right]$ are isometrically embedded onto the Klein disk,^43^ the planar coordinates of the vertices *v*_*i*_, *w*_*i*_ are denoted by the same symbol $v_{i}$, $w_{i}$. Then the Jacobian matrix for the derivative map dφ can be explicitly computed as^44^:

(1) $$J=\mathrm{d\phi}=\left[w_{3}-w_{1},w_{2}-w_{1}\right]{\left[v_{3}-v_{1},v_{2}-v_{1}\right]}^{-1}$$

Finally, the TBM is defined as $\sqrt{\det\left(J\right)}$. The TBM is complementary to the RD in that it captures localized changes in surface area.

Multivariate TBM (mTBM) is an enhancement of the TBM feature. It captures surface deformation perpendicular to the direction normal to the surface.^45^ It can be expressed as $\log\sqrt{JJ^{T}}$, which is a feature with dimension 3 × 1.

Our previous studies integrated RD and mTBM into the multivariate morphometry statistics (MMS).^11^ This work estimated MMS for each vertex on the individual hippocampal as a 4 × 1 vector. That is, each individual hippocampus can be represented as a *W* × 4 feature matrix, W is the vertices number of each hippocampus surface.

#### 2.4.3 Hippocampus surface MMS smoothing

The heat kernel smoothing algorithm was introduced to smooth the hippocampal surface features.^43^, ^46^ Referring to our previous work,^43^ key parameters of the heat kernel smoothing algorithm were set as: smoothing parameter σ=1 and number of iterations *m* = 10.

#### 2.4.4 Group‐wise hippocampus surface deformation analysis

The Hotelling's *T* ^2^ test was performed to evaluate the pair‐wise morphometric differences of the smoothed hippocampus surfaces in the HC‐AD, HC‐MCI, and MCI‐AD groups.^47^, ^48^ Statistical results were corrected for multiple comparisons by using the permutation test. We first calculated the group‐level hippocampal surface differences for each vertex as the ground true group using the Mahalanobis distance,^49^ then we performed 10,000 permutations of the alignment test. For each repetition, all samples were randomly divided into two groups and the Mahalanobis distance between groups was calculated. The probability (uncorrected p value) at each vertex was defined as the ratio of the random number of permutations with values greater than the ground truth group difference to the total number of permutations. Across all vertices, hippocampus morphometry group differences were shown in the form of *p* map. Thereafter, the *p* feature was defined as the number of vertices with uncorrected *p* values below a threshold (p<0.05), *p* features were the true effect in the real experiment. By comparing the actual *p* features with the 10,000 *p* features obtained from the random test, we obtained a ratio that describes the proportion of effects with similar or greater magnitude than the actual effect that occurred in the random assignment. This ratio was the chance that the observed pattern occurred by chance, and it provided the global significance level of the map, and we accepted the alignment p map if the ratio <0.05.^29^

#### 2.4.5 Effect size analysis

The previous study suggested that Cohen's *d* effect size method can determine the degree of hippocampus deformations in group 1 compared to group 2.^50^ Whereas MMS is a multivariate measure, Cohen's *d* effect size methods cannot be directly applied. The study pointed out that Mahalanobis distance can provide a multivariate measure of effect.^49^ Within the significant deformation subregions, we made vertex‐wise effect size statistics for HC‐AD, HC‐MCI, and MCI‐AD groups using the Mahalanobis distance, as in (2). Taking HC‐AD as an example, where $M_{1}$ and $M_{2}$ are the mean 4 × 1 MMS vector per hippocampus surface vertex of HC and AD groups respectively, *S* is their corresponding 4 × 4 covariance matrix. The Mahalanobis distance $D^{2}$ is the multivariate analog of the univariate Cohen's *d* effect size.^51^

(2) $$D^{2}={\left(M_{1}-M_{2}\right)}^{T}S^{-1}\left(M_{1}-M_{2}\right)$$

### 2.5 Hippocampus MMS feature reduction and machine learning classification

#### 2.5.1 Patch selection

After image processing and features extraction, there were 15,000 vertices on each side of the hippocampus surface, and each vertex is a MMS feature vector. That is, the feature dimension of single hippocampus of each subject was 60,000. It can be seen that the feature dimension is much larger than the number of subjects, i.e., high dimensional and small sample problem, so we adopted the following method to refine and downsample hippocampus features.

In the MMS software, the 15,000 vertices of each hippocampus were mapped into a matrix of [100, 150] according to the conformal parameters. In order to refine the features, we first employed rectangular windows (patches) on the mapped matrix to select surface vertex features by equally spaced sliding. Because larger effect sizes indicate stronger differentiation effects for different categories, based on the effect size hierarchy, we only selected patches with a mean effect size greater than 0.8 for subsequent classification. We calculated the average effect of each patch using the following method: we first calculated the effect sizes of each surface vertex for HC‐AD, HC‐MCI, and MCI‐AD groups using the Mahalanobis distance by (2), based on this, we calculated the average effect size of the three groups at each vertex and then calculated the average effect size for each patch (the average effect sizes of all vertices on each patch).

**FIGURE 2:**
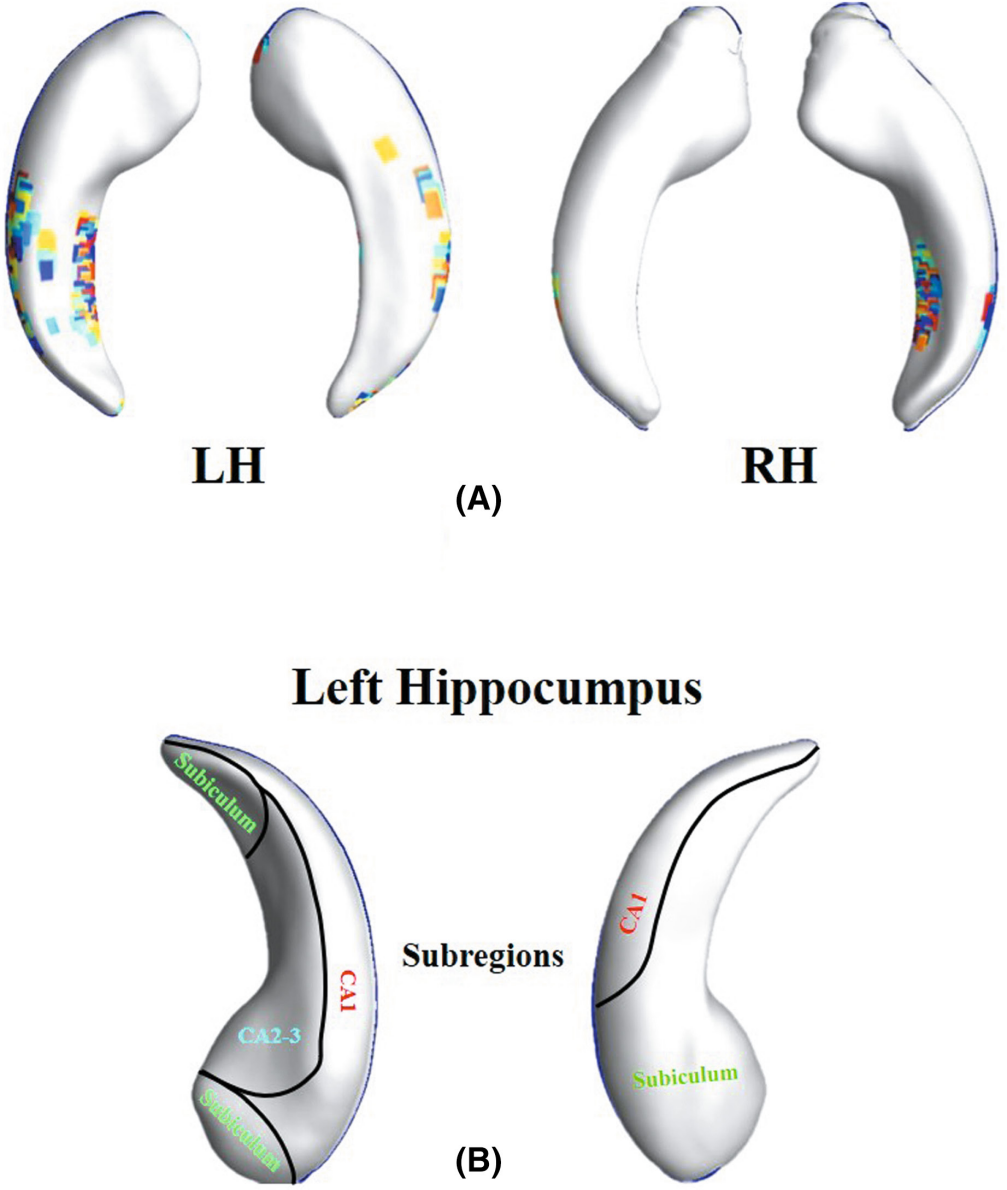
(A) Patches selected on the bilateral hippocampus surface. The colored rectangular blocks represent the final selected patches. Total selected 1886 patches on the left hippocampus and 627 patches on the right hippocampus, the patch size is: LH, left hippocampus; RH, right hippocampus. (B) Subregions of the left hippocampus template. The template of the hippocampus was subdivided into three different subregions, including CA1, CA2–3, and Subiculum.

We tried patches in turn, with 2 as the iteration interval, and finally found that the best performance was achieved with a patch size. Figure 2A visualizes the patches selected on the bilateral hippocampus surface, and we reshaped the MMS features contained in each patch into an m‐dimensional vector $x_{i}$, and all the vectors *x*_i_ constituted a matrix $X=\left(x_{1},x_{2},\ldots ,x_{n}\right)\in R^{m\times n},x_{i}\in R^{m},i=1,2,\ldots n$ by column, where *n* is the total number of patches.

#### 2.5.2 Patch‐based sparse coding and dictionary learning

To further improve calculation efficiency, we used online dictionary learning (ODL) to learn overcomplete dictionary by modeling the matrix X as a sparse linear combination of column vectors (atoms) in the learned dictionary D.^52^

According to ODL, the sparse coding optimization problem for each column vector $x_{i}$ in X can be expressed in the form of (3):

(3) $$\min_{D\in C}\;\frac{1}{n}\sum_{i=1}^{n}\min_{\alpha_{i}}\;\left(\frac{1}{2}{∥x_{i}-D\alpha_{i}∥}_{2}^{2}+\psi \left(\alpha_{i}\right)\right)\;$$

where n is the number of vectors of X, D is the learned overcomplete dictionary, each column represents a basis vector (atom) and tis the size of the dictionary. $\alpha_{i}$ is usually called sparse encoding. ψ is the sparse‐induced regularizer and C is the constraint set of the dictionary D. ψ and C can be used in various combinations to solve different matrix decomposition problems. To prevent arbitrary scaling of the sparse encoding, the atoms *d*_1_ of *D* are constrained to be $C≜\left\{D\in R^{m\times t}\right.$ s.t. $\left.\forall j=1,\ldots ,t,d_{j}^{T}d_{j}\leq 1\right\}$, and the regularizer ψ we use L1‐regularization, because it ensures that the learned features are sparse, i.e., $\;\alpha_{i}$ has only a few nonzero values.

Before applying the sparse coding, we constrained the input X by columns as L2 norm, and then we can rewrite (3) as a matrix decomposition problem as follows:

(4) $$\min_{D\in C,\;A\in R^{t\times n}}\;\frac{1}{2}∥X-\mathrm{DA}{∥}_{F}^{2}+\lambda ∥A{∥}_{1}\;$$

where $A=\left(\alpha_{1},\alpha_{2},\ldots ,\alpha_{n}\right)\in R^{t\times n}$,$\;\alpha_{i}\in R^{t}$, i=1,2,…n. Finally, we used the learned dictionary D to encode m‐dimensional feature vector $x_{i}$ into a t‐dimensional sparse encoding $\alpha_{i}$, i.e., we can use $x_{i}=D\alpha_{i}$ to approximate $x_{i}$.

We reshaped selected patches (effect size >0.8) of each subject's bilateral hippocampus after sparse encoding into a p‐dimensional vector $s_{i}$ by column, and $s_{i}$ is the set of all sparsely encoded hippocampus features of the subject, the feature vectors of all subjects constituted a sparse matrix $S=\left(s_{1},s_{2},\ldots ,s_{q}\right)\in R^{p\times q}$, where p is the final hippocampus feature dimension of each subject and q is the total number of subjects.

#### 2.5.3 Feature reduction

The hippocampus feature of each subject after sparse coding is a high dimension and sparse vector, and based on the highly sparse nature of the information, we employed independent‐samples *T*‐test to select features in *S* that are more significant for classification.

The *T*‐test‐based feature ranking and feature selection method is an effective method for high‐dimensional feature selection,^53^ which can test whether the mean of each feature of two independent samples is significantly different from its distribution (p<0.05).

We first divided all subjects into training and test sets and split all samples in test sets into three groups according to classes: HC‐MCI, HC‐AD, and MCI‐AD. The test statistic p value was then calculated for each feature in each of the three groups by using the independent samples *T*‐test, and the p value of all features was ranked in descending order. p value was chosen as an empirical value, due to the small number of subjects and high feature dimension, we only selected features with p value <0.01, so as to remove features with little difference (small difference between the two classes). We selected three groups of features that were significant for dichotomy in HC‐MCI, HC‐AD, and MCI‐AD. After that, we merged the three groups of features selected for binary classification and removed the duplicate features and use it as the features of the HC‐MCI‐AD triple classification group. We applied the feature indexes selected on the training set to the validation set and the test set for feature selection. To avoid overfitting of the model, we finally used Principal Component Analysis (PCA) dimension reduction to remove redundant information.

#### 2.5.4 Training classifier and parameter optimization

After a series of feature reduction processes, we finally obtained the low dimensional surface‐based hippocampal features for classification.

In this paper, we used SVM with a nonlinear kernel function for classification and built a triple SVM classifier using One‐Vs‐One multiclass strategy. One‐Vs‐One approach splits the dataset into one dataset for each class versus every other class and constructs one classifier per pair of classes. Each binary classification model may predict one class label and the class which received the most predictions are selected. In the event of a tie (among two classes with an equal number of votes), it selects the class with the highest aggregate classification confidence by summing over the pair‐wise classification confidence levels computed by the underlying binary classifiers. This method may be advantageous for algorithms such as kernel algorithms.

The parameter optimization of SVM classifier is also an important aspect. The main hyperparameter optimization methods commonly used are grid search, random search, and Bayesian optimization.^54^ Bayesian optimization was applied because of its more efficient than the other two search methods.^55^ Ten‐fold cross‐validation scheme was applied to evaluate the classification performance. The scheme was repeated a total of 10 times.

Finally, we measured the performances of kinds of classifiers. And the classification performance of hippocampal MMS features was compared with volume and age features. For binary classification, we used accuracy, sensitivity, specificity, and area under the curve (AUC) in receiver operating characteristic (ROC). In total, we tested three different binary classifications, HC‐AD, HC‐MCI, and MCI‐AD. For triple classification, we used standard performance metrics, precision, recall, F1‐score, accuracy, and confusion matrix. All the ML analyses were done using scikit‐learn, a python library for machine learning.^56^

### 2.6 Correlation analysis of hippocampus surface features and MMSE scores

The level of the Brief Mental Status Examination Scale (MMSE) scores reflects the subject's intellectual status and the degree of cognitive disability.^57^ To better reveal the association between hippocampal MMS with clinical MMSE measure, we made the Pearson correlation analysis between the MMSE scores and the MMS (RD and mTBM) features of each vertex separately for each subject.

The RD or mTBM features of all subjects at each vertex can be expressed as a matrix, where n is the number of subjects and m is the total number of vertices on each side of the hippocampus, as in (5):

(5) $$\left(\begin{array}{ccc} v_{1}^{1} & \cdots & v_{1}^{n}\\ \vdots & \ddots & \vdots \\ v_{m}^{1} & \cdots & v_{m}^{n} \end{array}\right)\;$$

Each subject has one MMSE score, which can be expressed as a vector of$\;\left[n,1\right]$, n is the number of subjects, as in (6):

(6) $$\left(\begin{array}{c} \mathrm{MMS}E_{1}\\ \mathrm{MMS}E_{2}\\ \vdots \\ \mathrm{MMS}E_{n-1}\\ \mathrm{MMS}E_{n} \end{array}\right)$$

We used vertex‐based Pearson correlation analysis, which is calculated by successively extracting each row of matrix (5) and doing Pearson correlation analysis with vector (6), which eventually generates a correlation coefficient (*p* value and *r* value) for each vertex on the bilateral hippocampus, and based on the correlation coefficient of each vertex, the regions with stronger correlations can be visualized on the bilateral hippocampus.

## 3 RESULTS

### 3.1 Study samples

Demographics and hippocampus volume information for the AD, MCI, and HC groups were summarized in Table 1. Chi‐Squared Test and Analysis of variance (ANOVA) were used to analyze the differences between categorical variables and numerical variables. ANOVA needs a normality test, we combine Shapiro–Wilk Test and histogram analysis. The results showed that the age distribution conforms to the absolute normal distribution, the hippocampal volume, CDR and MMSE were approximately normally distributed. ANOVA has a certain tolerance to the normality of the data, as long as the data is approximately normal, so all numerical variables meet the requirements of ANOVA. There were significant differences in age, hippocampal volume, CDR and MMSE among the three groups by using ANOVA.

**TABLE 1:** Participant demographics and hippocampus volume information.

	HC	MCI	AD	Normality test	Test statistics
Sample size	102	63	75	–	–
Age, mean (SD)	64.9 (7.7)	69.2 (7.8)	65.6 (8.9)	Absolute normal	*F* = 5.9; *p* = 0.003
Male/female	46/56	23/40	31/44	–	*χ* ^2^ = 1.187; *p* = 0.552
LH volume (cm^3^), mean (SD)	5176.3 (577.2)	4625.8 (846.5)	4059.2 (860.3)	Approximate normal	*F* = 48.4; *p* = 0.000
LH volume (cm^3^), mean (SD)	4885.1 (811.0)	3981.5 (1244.0)	3779.4 (975.5)	Approximate normal	*F* = 31.4; *p* = 0.000
CDR, mean (SD)	27.26 (3.22)	24.08 (3.66)	14.68 (6.18)	Approximate normal	*F* = 830.2; *p* = 0.000
MMSE, mean (SD)	0.00 (0.00)	0.51 (0.06)	1.43 (0.53)	Approximate normal	*F* = 177.9; *p* = 0.000

Abbreviations: AD, Alzheimer's disease; HC, healthy control; LH, left hippocampus; MCI, mild cognitive impairment; RH, right hippocampus; SD, standard deviation.

### 3.2 Hippocampus morphometry analysis

#### 3.2.1 Hippocampus subregions definition

According to previous study related to hippocampus subregions,^21^ the template of the hippocampus was subdivided into three different subregions, mainly including CA1, CA2‐3, and Subiculum (Figure 2B).

#### 3.2.2 Hippocampus morphology differences

**FIGURE 3:**
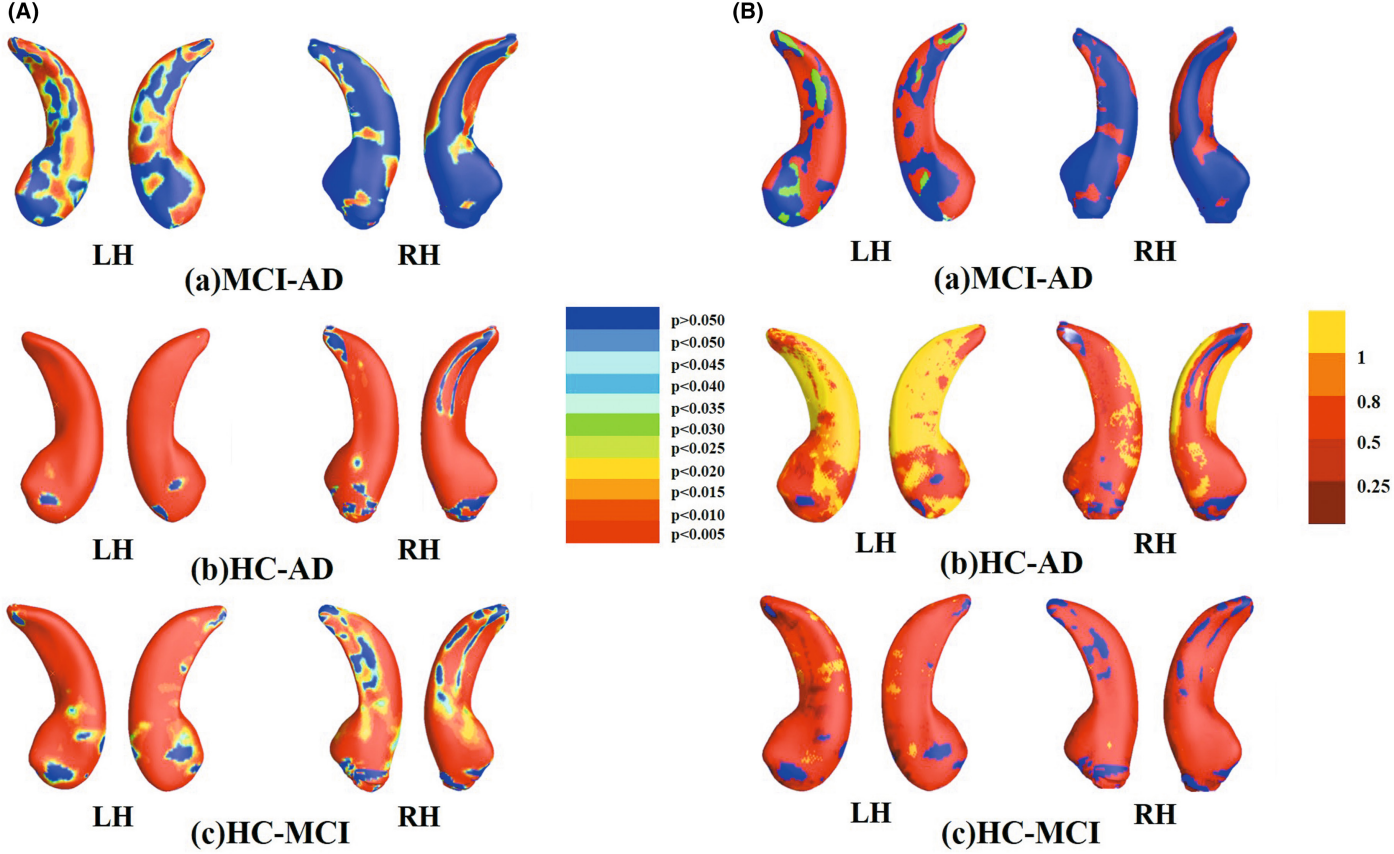
(A) Group difference maps of the bilateral hippocampus in the HC‐MCI, HC‐AD, and MCI‐AD groups. After we used 10,000 permutation test, nonblue color indicates that the vertices of the region with statistical differences at the nominal 0.05 level, uncorrected for multiple comparisons. (B) MMS effect size maps for the bilateral hippocampus vertices of the bilateral hippocampus in the HC‐MCI, HC‐AD, and MCI‐AD groups. Larger effect sizes indicated higher differentiation between the two groups. The highest effect size was located in the left hippocampus CA1 region in the HC‐AD group, most regions in both the HC‐AD and HC‐MCI groups had effect sizes greater than 0.8 (red), and the left hippocampus CA1 region in the HC‐AD group had a higher effect size (yellow).

Significant morphology differences were found in the whole bilateral hippocampus, including CA1, CA2/3, and Subiculum in the HC‐MCI and HC‐AD groups. For the MCI‐AD group, the distribution of significant differences regions in the bilateral hippocampus was slightly different, with significant differences in the left hippocampus mainly in the CA1 region and the local area of the subiculum, and significant differences in the right hippocampus mainly in the CA1 region (Figure 3A).

#### 3.2.3 Effect size analysis

From Figure 3B, there were significant differences of effect size of MMS between HC‐AD, HC‐MCI, and MCI‐AD groups. The larger effect sizes indicated higher differentiation between the two groups.

### 3.3 Classification performance evaluation

#### 3.3.1 Binary classification

**FIGURE 4:**
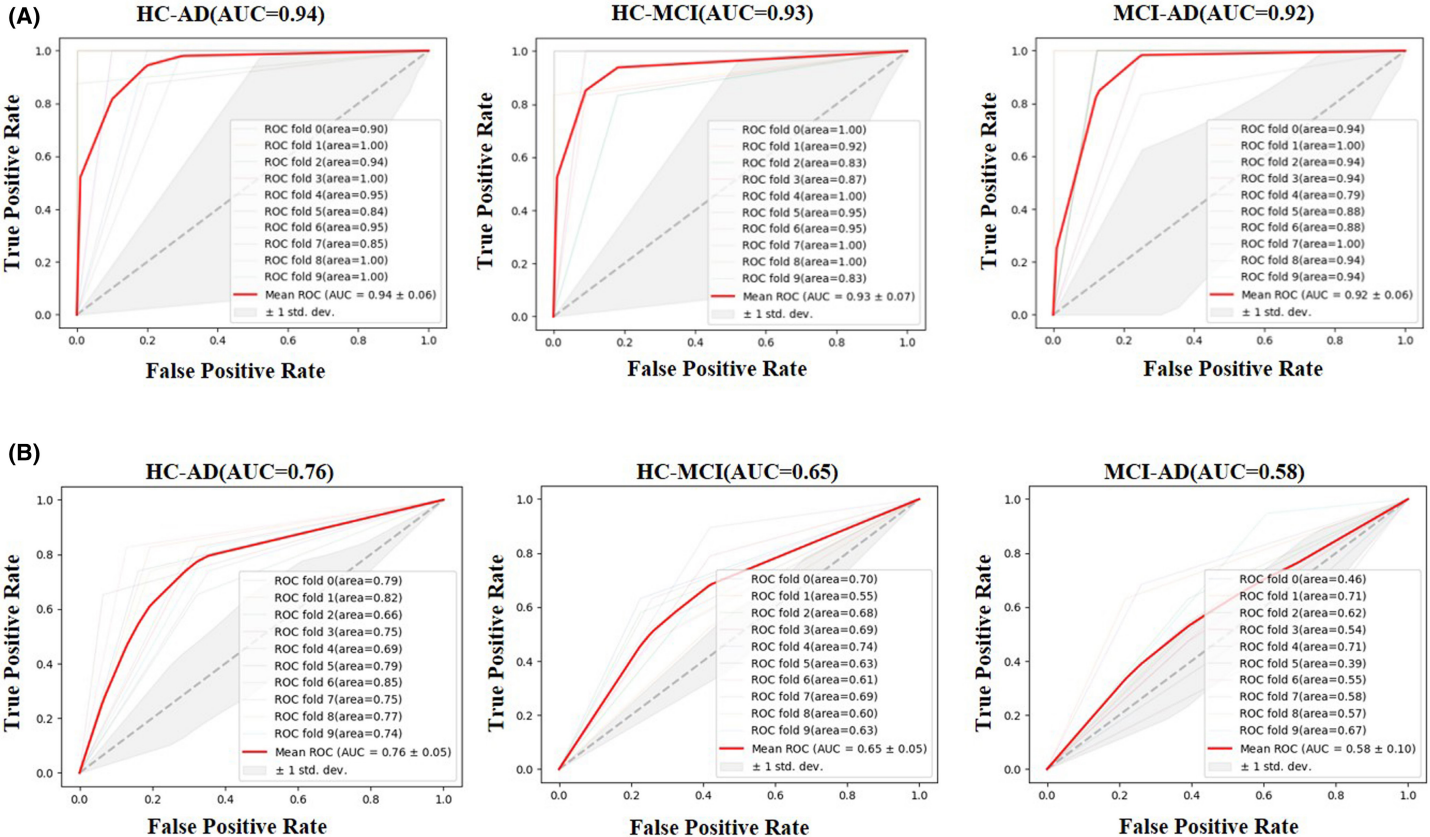
AUC performance of binary classifiers by ten‐fold cross‐validation. AUC, area under curve. (A) ROC result using hippocampal MMS features (B) ROC results using hippocampal volume and age features.

Figure 4 showed the performance of binary classification of HC‐AD, HC‐MCI, and MCI‐AD by ten‐fold cross‐validation and receiver operating characteristic curve (ROC) analysis. It can be clearly seen that the AUC was higher in the three groups classified using hippocampal MMS features. As the results, the classification of HC‐AD group showed the best performance, with an area under curve (AUC) of 0.94, and the classification of HC‐MCI and MCI‐AD groups also showed good performance, with AUC of 0.93 and 0.92, respectively (Figure 4A and Table 2). Figure 4B and Table 2 showed the ROC results using hippocampal volume and age features (AUC of the classification of HC‐AD, HC‐MCI, and MCI‐AD was 0.76, 0.66, and 0.58, respectively).

**TABLE 2:** Performances of binary classifier by 10‐fold cross‐validation.

	HC‐AD	HC‐MCI	MCI‐AD
Accuracy	0.94 (0.76)^a^	0.93 (0.66)	0.92 (0.58)
Sensitivity	0.93 (0.76)	0.93 (0.68)	0.92 (0.54)
Specificity	0.94 (0.76)	0.92 (0.63)	0.92 (0.62)

Abbreviations: AD, Alzheimer's disease; HC, healthy control; MCI, mild cognitive impairment.

^a^ The classification results using hippocampal volume and age characteristics were shown in the parentheses.

#### 3.3.2 Triple classification

**FIGURE 5:**
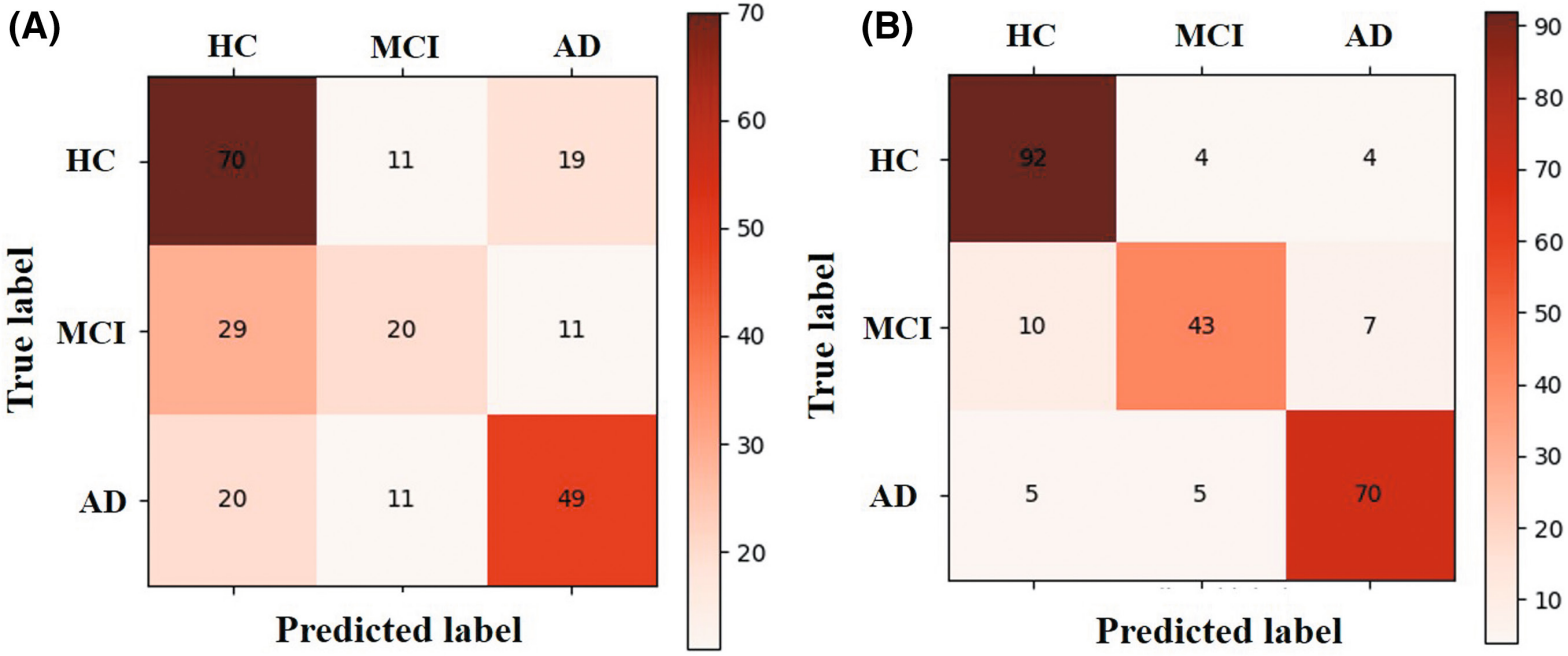
Triple classification result for 240 subjects with 10‐fold cross‐validation. The horizontal axis represents the predicted classes and the vertical axis represents the true classes, with larger diagonal values and darker colors indicating higher classification accuracy. (A) confusion matrix result using hippocampal volume and age feature. (B) confusion matrix result using hippocampal MMS features.

In Figure 5 and Table 3, the classification performance of each group was evaluated, it can be clearly seen that the classification results using hippocampal MMS features are better than the classification results using the hippocampal volume and age features. For HC, when using the hippocampal MMS features, the classifier achieved a better precision, recall, f1‐score with 0.86, 0.92, and 0.89, respectively. For MCI, the result was 0.83, 0.72, and 0.77, respectively. For AD, the result was 0.86, 0.88, and 0.87, respectively. Briefly, the overall accuracy of classification of the three groups reached 0.85, which was much higher than the accuracy of using hippocampus volume and age as the classification features (0.58).

**TABLE 3:** Classification performance of each class by 10‐fold cross‐validation.

Class	Precision	Recall	F1‐score
HC	0.86 (0.59)^a^	0.92 (0.70)	0.89 (0.64)
MCI	0.83 (0.48)	0.72 (0.33)	0.77 (0.40)
AD	0.86 (0.62)	0.88 (0.61)	0.87 (0.62)
Accuracy			**0.85 (0.58)**

*Note*: The bold value refers to overall accuracy, that is, the proportion of correctly predicted samples in all test samples.

Abbreviations: AD, Alzheimer's disease; HC, healthy control; MCI, mild cognitive impairment.

^a^ The classification results using hippocampal volume and age characteristics were shown in the parentheses.

### 3.4 Correlation analysis

**FIGURE 6:**
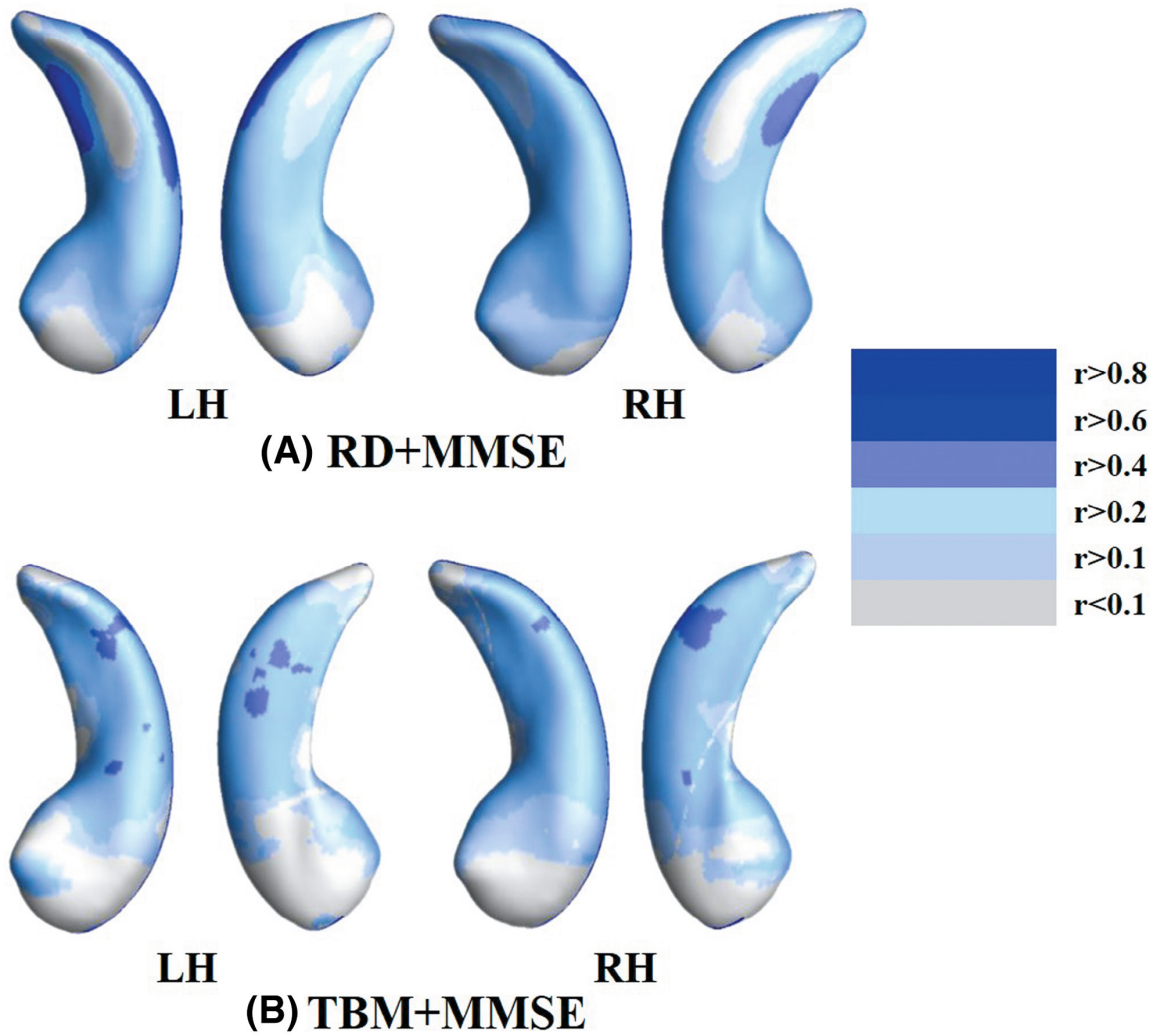
The association between hippocampal MMS with clinical MMSE measure. (A) Correlation areas of hippocampus in radial distance (RD). (B) Correlation areas of hippocampus in tensor‐based morphometry (TBM). A larger r indicates a higher correlation, which is represented as a darker blue in the color bar. LH, left hippocampus; RH, right hippocampus.

Positive correlations were revealed between hippocampus RD/TBM features and MMSE scores. The presence of regions of positive correlation with MMSE scores mainly included the CA1 region, then the CA2–CA3 region and the subiculum at a moderate level (0.4 < *r* < 0.6), the details see Figure 6.

## 4 DISCUSSION

### 4.1 Major findings

The present study showed significant deformation of hippocampus subregions during the transformation from HC to MCI/AD using MMS, which was closely associated with cognitive performance. Additionally, when the hippocampal MMS features were used to construct the binary classification and triple classification model, relatively good classification effect was achieved.

### 4.2 Hippocampal morphometric differences of AD vs. MCI vs. HC

Previous MRI studies reported significant structural changes in several brain regions in AD and MCI patients, including the whole‐brain,^58^ hippocampus,^29^, ^51^, ^59^ entorhinal cortex,^60^ ventricular expansion,^11^, ^61^ and temporal lobe volumes,^62^ which were closely related to cognitive function. Among these regions, the hippocampus formation was the most sensitive region for the AD pathology, which was vulnerable to amyloid protein deposition, hypoxia, ischemia, and so on.^63^, ^64^

The hippocampus, composed of the CA1, CA2/3, and subiculum, was important for memory storage and retrieval. In particular, the CA1 subregion constituted the primary output of the hippocampus, which was thought to be essential for most hippocampus‐dependent memories.^65^, ^66^ In the present study, we found that the deformation of CA1 region was more significant during the transformation of MCI to AD, which was consistent with the previous studies.^29^ In addition, interestingly, we found the deformation of the left hippocampus was more extensive than that of the right hippocampus, which was consistent with the previous studies.^67^, ^68^ Foley revealed that the onset of left hippocampus atrophy was earlier than that of the right hippocampus in 272 young healthy adults with higher polygenic risk scores. Moon demonstrated that the left hippocampus had a faster volume reduction than that of the right side in subjects of APOE‐e4 carriers. In addition, a previous study revealed that the left and right hemispheres exhibited functional differences, for instance, the left one was dominant for verbal cognitive function, and the right one was dominant for the spatial cognitive function,^69^ suggesting the decline of different cognitive functions during the gradual progression from HC to MCI and AD. This interesting finding needs to be further discussed and verified in future studies.

### 4.3 Binary and triple classification model construction at the individual level based on the hippocampal MMS features

Most previous studies focused on the hippocampal structural differences at the group level, especially between two groups.^11^, ^29^, ^70^ This is the first study where surface‐based hippocampal morphology measure was used to differentiate among three groups, including AD, MCI, and HC, and to classify individual subjects into diagnostic groups.

In this study, all the binary classification models based on hippocampal MMS features showed good performances, particularly presenting an AUC of 0.94 in the classification of HC‐AD group. In one of our previous studies, based on the whole brain gray matter voxel as feature, the accuracy of the binary classification model on AD/HC was 0.90.^71^ The current study was superior to the previous study, indicating that hippocampal MMS features could be used as better imaging indicators for AD, MCI, and HC pairwise classification.

In this study, the accuracy of the triple classification model based on hippocampus deformation reached 0.85. From Table 3, this classifier of HC achieved the best precision followed by AD, and then MCI. It could be explained as follows: due to the transitional stage of MCI between AD and HC, MCI was easier to be confused with AD or HC, which might influence the accuracy of the triple classification.

### 4.4 Correlation analysis of RD/TBM features of hippocampus surface vertex and MMSE scales

The correlation analysis showed that MMSE scores and hippocampus morphology feature (RD/TBM) values were positively correlated, i.e., the higher the MMSE, the more normal the hippocampus morphology, suggesting that the deformation of hippocampus could be used as an imaging marker for tracking disease progression. In addition, the area of positive correlation was mainly distributed in the CA1 region, followed by the CA2/3 region and the subiculum, which was consistent with the above result of hippocampus deformation subregions, further verifying the reliability and accuracy of using deformation characteristics of hippocampal subregions as markers for tracking AD progression.

## 5 LIMITATIONS

There are some limitations to our study. First of all, this is a cross‐sectional study. In the future, multi‐center longitudinal large sample data need to be collected and performed to confirm the current results. Second, to explore whether the surface‐based hippocampal morphometry measure could discriminate AD patients in different stages, future studies will add more samples of early stages of AD patients, such as ApoE 4 carriers and subjective cognitive decline. Finally, in this study, we only focused on the surface‐based hippocampus morphometry. Future work will need to be performed on other brain regions, such as the entorhinal cortex, amygdale, posterior cingulate cortex, and so on.

## 6 CONCLUSIONS

In conclusion, the present study found that significant deformation of hippocampus subregions occurred during the transformation from a healthy state to MCI or AD. Additionally, these changes were closely associated with cognitive performance. Most important of all, the good performance of the binary classification and triple classification models using the hippocampal MMS features confirmed that hippocampal MMS could be used as sensitive imaging biomarkers for the early diagnosis of AD at the individual level.

## AUTHOR CONTRIBUTIONS

Weimin Zheng and Honghong Liu were responsible for the acquisition, analysis, and interpretation of data and drafting of the manuscript; Zhigang Li was responsible for part of the data analysis; Kuncheng Li and Yalin Wang were responsible for the clinical function assessment of patients; Bin Hu, Qunxi Dong, and Zhiqun Wang were responsible for the final approval of the version of the manuscript to be published.

## FUNDING INFORMATION

This work was supported by the National Natural Scientific Foundation of China (81873892), Natural Science Foundation of Beijing Municipality (7222320), Capital Health Research and Development of Special Fund (2022–2‐6081), Scientific Research Fund of Aerospace Center Hospital (YN201901), the Fundamental Research Funds for the Central Universities (lzujbky‐2021‐kb26) and Beijing Institute of Technology Research Fund Program for Young Scholars.

## CONFLICT OF INTEREST STATEMENT

The authors declare no conflicts of interest.

## Data Availability

The raw/processed data during the study are proprietary and confidential, it cannot be shared at this time as the data also forms part of an ongoing study.
